# Association between physical exercise based on FITT principle and vision health among primary school students in Suzhou, China

**DOI:** 10.3389/fpubh.2026.1797792

**Published:** 2026-05-11

**Authors:** Miyu Wang, Gang Chen, Xing Wang, Guiming Zhu, Kaixin Niu, Sheng Zhou, Rongbin Yin

**Affiliations:** 1 Physical Education and Sports School, Soochow University, Suzhou, China; 2 Department of Basic Course, Suzhou City University, Suzhou, China

**Keywords:** FITT principle, physical exercise, primary school students, vision health, dose–response

## Abstract

**Objective:**

This study aimed to examine the association between physical exercise based on the FITT principle (frequency, intensity, time, and type) and vision health, including uncorrected distance visual acuity (UDVA) and kinetic visual acuity (KVA) among Chinese primary school students.

**Methods:**

A total of 1,649 students from grades 2 to 5 across five primary schools in Suzhou, China, were selected as the study participants. Information on physical exercise, including frequency, intensity, time, and type, was collected using structured questionnaires. Exercise volume was additionally calculated to reflect overall physical activity exposure. Data on eye usage habits, visual environments, and outdoor activity time were also collected. UDVA and KVA were assessed using a standard logarithmic visual acuity chart and a kinetic visual acuity tester, respectively. Generalized Propensity Score Matching (GSPM) was employed to estimate the associations between exercise frequency, intensity, time, volume, and the UDVA and KVA of the students. Additionally, the traditional Propensity Score Matching (PSM) was applied to examine the associations between different types of exercise and the students’ UDVA and KVA.

**Results:**

No significant gender difference was found in UDVA (*p* > 0.05), whereas KVA varied significantly by gender (*p* < 0.05). Additionally, both metrics showed significant differences across grade levels (*p* < 0.05). The associations between exercise frequency, intensity, time, volume, and the students’ UDVA and KVA exhibited an “inverted U-shaped” pattern. Moreover, participation in open-skill sports was associated with more favorable UDVA compared with closed-skill sports.

**Conclusion:**

Among students in grades 2 to 5 in Suzhou, UDVA declined progressively with increasing grade level, while KVA peaked in grade 4 and subsequently declined. The more positive associations with both UDVA and KVA were observed among primary school students engaging in physical exercise 3 to 4 times per week at moderate intensity and moderate volume. Exercise durations of 61 to 90 min per session and 91 to 120 min per session showed the more significant correlation with higher levels of UDVA and KVA, respectively. Additionally, open-skill sports were more significantly associated with the healthy development of UDVA compared to closed-skill sports.

## 1 Introduction

Myopia has emerged as a global public health challenge affecting children and adolescents worldwide. According to the World Health Organization’s Vision Report (1), over 2.6 billion people globally are affected by myopia, with approximately 312 million (12%) being individuals under 19 years of age. Projections suggest that by 2050, the global prevalence of myopia and high myopia will increase substantially, affecting approximately 50 and 10% of the world’s population, respectively (2). This trend toward earlier onset of myopia is particularly concerning; for instance, an epidemiological study in Northern Ireland reported that the myopia prevalence is 1.9% among children aged 6–7 years and 16.4% among those aged 12–13 years—more than double the rate observed in the same age group 50 years ago (3). The highest myopia prevalence rates are currently observed in high-income Asia-Pacific countries (53.4%), closely followed by East Asia (51.6%), with particularly high rates reported among urban adolescents in these regions (2). China, as one of the most affected countries in the East Asian region, faces a significant myopia challenge. According to the latest monitoring data released by the National Bureau of Disease Control and Prevention (2024), the overall prevalence of myopia among Chinese children and adolescents reached 51.9% in 2022. The prevalence showed a progressive increase with educational level—36.7% in primary school, 71.4% in junior high school, and 81.2% in senior high school (4). The widespread prevalence of myopia not only impairs students’ academic performance and social development but also restricts their long-term developmental potential (5), posing a significant challenge to the implementation of the “Healthy China” strategy.

As a fundamental ability for individuals to engage in learning and daily life, visual function is influenced by a variety of factors. From a physiological perspective, the primary cause of refractive errors lies in prolonged near work, which leads to sustained contraction of the ciliary muscle. This, in turn, affects the regulation of the lens shape and induces axial elongation of the eyeball (6). The ciliary muscle, as a key component of the visual accommodation system, enables rapid switching between near and distant focus primarily by altering the curvature of the lens. Specifically, when the eye focuses on a near object, the ciliary muscle contracts, releasing tension in the zonular fibers and allowing the elastic lens to become more convex, thereby increasing its refractive power. Conversely, when focusing on a distant object, the ciliary muscle relaxes, the lens flattens, and distant focus is achieved (7). Prolonged near work results in sustained contraction of the ciliary muscle, imposing excessive demands on the accommodative system. This may lead to muscle fatigue and accommodative lag, ultimately causing abnormalities in lens shape regulation and blurred vision. If this condition persists, the accommodative response of the ciliary muscle may decline, and the accommodative lag can further promote axial elongation, which is a key contributing factor in the development of axial myopia.

Kinetic visual acuity (KVA) refers to the ability of the eyes to maintain clear visual recognition while tracking fast-moving objects or targets with continuously changing distances. This visual function is particularly important for children in daily activities such as classroom learning and ball games, where target tracking and spatial judgment are essential (8). In contrast, uncorrected distance visual acuity (UDVA) primarily assesses the ability to distinguish static objects at a distance under non-moving conditions. While KVA and UDVA relate to different visual contexts and rely on distinct accommodative mechanisms, they are closely interconnected at the physiological level. The realization of KVA depends on the ciliary muscle’s ability to rapidly adjust focus in response to changes in target distance. This frequent near-to-far visual switching process serves as effective training for the flexibility and responsiveness of the ciliary muscle. Although UDVA is assessed under static conditions, its maintenance also relies on the stable tension and accommodative function of the ciliary muscle (9). Thus, despite their differing functional roles, both KVA and UDVA share a common dependence on the ciliary accommodation system. Previous research has indicated that visual training involving repeated stimulation of near-far focus adjustment (e.g., dynamic visual tracking exercises) can significantly enhance KVA while also improving the endurance and sensitivity of ciliary accommodation, thereby indirectly contributing to improvements in UDVA (10). Moreover, KVA has been identified as a crucial mediating factor in the process by which physical activity promotes visual health in children and adolescents (11). These findings suggest that KVA and UDVA are not independent functions, but rather work synergistically to support ocular development and maintain visual health.

In recent years, increasing attention has been paid to the potential role of physical activity in vision health. Early research primarily focused on the benefits of physical exercise for cardiopulmonary endurance and overall physical fitness (12–14). With growing interest in visual health, subsequent studies have begun to explore the relationship between physical activity and visual function, particularly in terms of static visual acuity and accommodative ability (15). More recent evidence suggests that physical exercise may also influence visual function by promoting periodic contraction and relaxation of the ciliary muscle, which may help maintain accommodative function and alleviate visual fatigue associated with prolonged near work (16). During physical activity, individuals often shift their gaze between near and distant targets, a process that resembles accommodative training used in clinical settings. This dynamic visual engagement may contribute to improved flexibility of the visual system. Furthermore, open-skill sports, which require continuous visual tracking, rapid focusing, and spatial judgment, may provide additional stimulation to the visual system. Such high-frequency visual demands have been suggested to produce effects comparable to dynamic accommodative training, thereby offering potential benefits for visual regulation and eye health (17, 18).

However, existing studies, both in China and internationally, have rarely examined the relationship between multidimensional physical activity characteristics—such as frequency, intensity, duration, and type—and vision health in a comprehensive manner. In particular, evidence regarding the optimal “dose” of physical activity for promoting visual function in children remains limited. Against this backdrop, the present study aims to examine the associations between physical activity characteristics based on the FITT principle (Frequency, Intensity, Time, and Type) and both uncorrected distance visual acuity and kinetic visual acuity in children. Additionally, the overall volume of physical activity—derived from the combination of frequency, intensity, and time—was evaluated to provide a comprehensive assessment. Furthermore, this study seeks to identify physical activity patterns associated with more favorable vision health outcomes, thereby providing empirical evidence to inform public health perspectives on physical activity and vision health in children and adolescents.

## 2 Research subjects and methods

### 2.1 Participants

**Figure 1:**
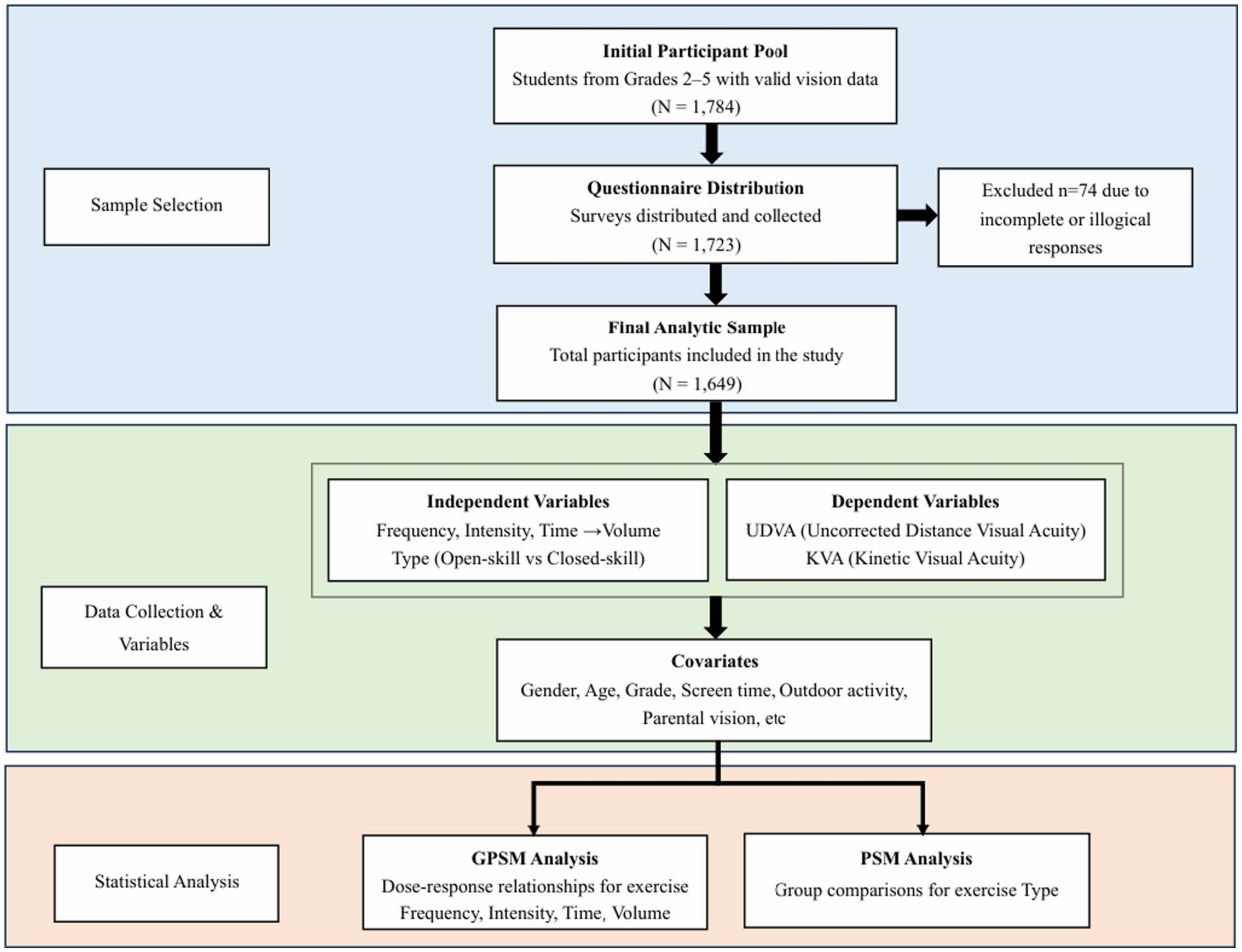
Study design and participant flowchart.

A total of 1,784 students from grade 2 to 5 were randomly selected using cluster sampling from 40 classes across five primary schools in the High-Tech Zone of Suzhou, China. The mean age of the participants was 9.39 ± 1.35 years. Uncorrected distance visual acuity and kinetic visual acuity were measured for all participants. A corresponding number of questionnaires were distributed, and 1,723 were returned, resulting in a response rate of 96.58%. After excluding responses with inconsistencies or missing data, 1,649 valid questionnaires were retained for analysis, yielding an effective response rate of 95.71%. The detailed research design and participant recruitment flowchart are illustrated in Figure 1. The demographic distribution of participants is presented in Table 1.

**Table 1:** Basic demographic information of survey participants.

Variable	Category	N	Percentage (%)
Age	7	133	8.1
	8	359	21.8
	9	376	22.8
	10	351	21.3
	11	376	22.8
	12	54	3.3
Gender	Male	855	51.8%
	Female	794	48.2%
Grade	Grade 2	415	25.2%
	Grade 3	407	24.7%
	Grade 4	418	25.3%
	Grade 5	409	24.8%

### 2.2 Questionnaire design and data collection

The questionnaire was designed based on the study’s required variables, including treatment variables, outcome variables, and covariates. It comprised three parts:

Part I: Demographic information, including name, gender, age, class, and school.

Part II: Covariates, covering individual (e.g., reading and writing time, outdoor activity time, screen time, continuous eye use time), family (e.g., parental vision status, family economic level, highest parental education level, parental academic expectations, extent of restrictions on the use of electronic devices at home, number of books at home), and school factors (e.g., homework load, the intensity of health education provided by the school).

Part III: Physical Exercise, focusing on exercise frequency, intensity, time, and type. Exercise frequency was categorized into five levels: Almost Never, 1–2 times/week, 3–4 times/week, 5–6 times/week, and ≥6 times/week (assigned scores of 1 to 5, respectively). Exercise intensity was measured across five levels: Very Low, Low, Moderate, High, and Very High (assigned scores of 1 to 5, respectively). Exercise time per session was divided into five intervals: 0–30 min, 31–60 min, 61–90 min, 91–120 min, and ≥120 min (assigned scores of 0 to 4, respectively). In this study, exercise volume was assessed based on frequency, intensity, and time, calculated as followed: Exercise Volume = Intensity × Time × Frequency. The classification criteria for exercise volume were as follows: ≤19: Low exercise volume; 20–42: Moderate exercise volume; ≥43: High exercise volume.

The reliability of the questionnaire was assessed via the test–retest method. Specifically, the designed questionnaire was first administered to 126 students, and after a two-week interval, the same questionnaire was administered again to these participants. Pearson correlation analysis of the two sets of results showed the following correlations: the partial correlation between physical exercise and vision-related factors was r = 0.801, and the partial correlation between exercise frequency, intensity, time, and type was r = 0.824. These results indicated that the reliability of the questionnaire is good.

### 2.3 Visual acuity measurement

To ensure the accuracy of the study, the inclusion criteria for participants in the visual acuity measurements were as follows:

1. No severe organ structural diseases, and no pathological eye conditions such as astigmatism, amblyopia, or keratitis.
2. Normal learning ability, motor skills, and operational ability.
3. No use of orthokeratology lenses (OK lenses).

UDVA was measured using the standard Logarithmic Visual Acuity Chart (GB11533–2011), under uniform lighting conditions and following the “National Student Physical Health Survey and Testing Guidelines.” The specific procedure was as follows: A marker was placed 5 meters away from the lightbox, and the participant stood upright behind the marker. The visual acuity of the left and right eyes was measured sequentially. During measurement, the untested eye was covered with an eye patch. If the participant wore corrective glasses, they were asked to remove the glasses and take a short break before proceeding with the uncorrected visual acuity measurement. In this study, the lower visual acuity value of the left and right eyes was used as the student’s UDVA.

KVA was measured using the XP.14-TD-J905 Kinetic Visual Acuity Tester (manufactured by Shanghai Tuofeng Automation Technology Co., Ltd.). During the measurement, the participant sat in the designated position in front of the device, maintaining an upright posture and keeping both eyes close to the viewing hole, looking inward. The device displayed a “C”-shaped visual target, which moved at a constant speed from far to near within the participant’s visual field. The opening direction of the “C”-shaped target could be upward, downward, leftward, or rightward. The participant was required to immediately push a joystick in the direction of the “C” opening they saw to complete the test. Each participant underwent three tests, with a 30-s interval between each, and the average of the three measurements was taken as the final KVA. All tests were conducted by the same examiner to ensure high objectivity and accuracy of the data. The test value range was 0.1 to 1.6, with higher values indicating better KVA.

### 2.4 Statistical analysis

Data were analyzed using SPSS 25.0 and STATA 16SE. The Kolmogorov–Smirnov test was conducted to assess the normality of UDVA and KVA data. Normally distributed data were described using means and standard deviations. Independent sample t-tests, one-way ANOVA, and post-hoc multiple comparisons were used to analyze differences in UDVA and KVA. A significance level of *p* < 0.05 was considered statistically significant. Generalized Propensity Score Matching (GPSM) was used to mitigate the influence of potential confounding variables, allowing for a more robust estimation of the dose–response relationships between exercise frequency, intensity, time, volume, and vision health (UDVA and KVA). Dose–response relationship graphs were plotted. Additionally, traditional Propensity Score Matching (PSM) was employed to examine the associations between various types of exercise and students’ UDVA and KVA.

## 3 Research results

### 3.1 Gender and grade differences in UDVA and KVA in students

Table 2 showed that there was no significant difference in UDVA between male and female students (*p* > 0.05), although males tad slightly higher scores. However, a statistically significant difference was observed in KVA (*p* < 0.05), with male students exhibiting better KVA levels than females. Given the large sample size (N = 1,649), these parametric comparisons remain robust according to the Central Limit Theorem, even though the raw KVA data show a skewed distribution.

**Table 2:** Visual acuity levels by gender.

Mean ± SD	Overall (*n* = 1,649)	Male (*n* = 855)	Female (*n* = 794)	95%CI		*t*-test	
				Lower	Upper	*t*	*p*
UDVA $\left(\bar{X}\pm S\right)$	4.92 ± 0.23	4.92 ± 0.23	4.91 ± 0.24	−0.012	0.034	0.958	0.338
KVA $\left(\bar{X}\pm S\right)$	0.56 ± 0.23	0.58 ± 0.23	0.55 ± 0.23	0.007	0.052	2.616	0.009

As shown in Tables 3,4, one-way ANOVA and post-hoc multiple comparisons were used to analyze the differences in UDVA and KVA across different grade levels. The results indicated that there was a significant difference in UDVA among students from different grades (*p* < 0.05). Specifically, second-grade students had significantly better UDVA than students from other grades, while fifth-grade students had significantly lower UDVA. Additionally, there was a significant difference in KVA across grade levels (p < 0.05), with fourth-grade students showing significantly better KVA than students from other grades.

**Table 3:** Visual acuity levels by grade.

Variable	Source	*n*	$\bar{X}\pm S$	Sum of Squares	DF	Mean Squares	*F* value	Sig.
UDVA	Grade 2	415	4.99 ± 0.17					
	Grade 3	407	4.96 ± 0.17					
	Grade 4	418	4.87 ± 0.28					
	Grade 5	409	4.85 ± 0.27					
	Total	1,649	4.92 ± 0.23					
	Between Groups			5.757	3	1.919	36.882	0.000
	Within Groups			85.592	1,645	0.052		
	Total			91.349	1,648			
KVA	Grade 2	415	0.50 ± 0.21					
	Grade 3	407	0.53 ± 0.22					
	Grade 4	418	0.63 ± 0.25					
	Grade 5	409	0.58 ± 0.20					
	Total	1,649	0.56 ± 0.23					
	Between Groups			4.732	3	1.577	31.391	0.000
	Within Groups			82.652	1,645	0.050		
	Total			87.384	1,648			

**Table 4:** *Post-hoc* comparisons of visual acuity among grades.

Dependent variable	(I) grade	(J) grade	Mean difference (I-J)	Std. error	Sig. (P)	95% CI lower	95% CI upper
UDVA	Grade 2	Grade 3	0.029	0.016	0.399	−0.013	0.071
		Grade 4	0.123*	0.016	0.000	0.082	0.165
		Grade 5	0.138*	0.013	0.000	0.096	0.180
	Grade 3	Grade 2	−0.029	0.013	0.399	−0.071	0.013
		Grade 4	0.094*	0.013	0.000	0.052	0.136
		Grade 5	0.108*	0.016	0.000	0.066	0.151
	Grade 4	Grade 2	−0.123*	0.016	0.000	−0.165	−0.082
		Grade 3	−0.094*	0.016	0.000	−0.136	−0.052
		Grade 5	0.014	0.016	1.000	−0.028	0.056
	Grade 5	Grade 2	−0.138*	0.016	0.000	−0.180	−0.096
		Grade 3	−0.108*	0.016	0.000	−0.151	−0.066
		Grade 4	−0.014	0.016	1.000	−0.056	0.028
KVA	Grade 2	Grade 3	−0.028	0.016	0.532	−0.068	0.016
		Grade 4	−0.137*	0.016	0.000	−0.178	−0.096
		Grade 5	−0.087*	0.016	0.000	−0.128	−0.045
	Grade 3	Grade 2	0.027	0.016	0.532	−0.015	0.068
		Grade 4	−0.111*	0.016	0.000	−0.152	−0.070
		Grade 5	−0.060*	0.016	0.001	−0.101	−0.019
	Grade 4	Grade 2	0.137*	0.016	0.000	0.096	0.178
		Grade 3	0.111*	0.016	0.000	0.070	0.152
		Grade 5	0.051*	0.016	0.007	0.010	0.092
	Grade 5	Grade 2	0.087*	0.016	0.000	0.045	0.128
		Grade 3	0.060*	0.016	0.001	0.019	0.101
		Grade 4	−0.051*	0.016	0.007	−0.092	−0.010

*p < 0.05.

### 3.2 Association between physical exercise and visual health in students

Table 5 presents the distribution of participants across different physical exercise levels, categorized by the FITT dimensions (frequency, intensity, time, and type). Regarding frequency, the majority of students (43.7%) participated in physical exercise 1 to 2 times per week, while only 4.1% engaged in exercise more than 6 times per week. In terms of intensity, participation was most concentrated at the moderate level (28.0%), whereas very high-intensity exercise was the least common, reported by only 1.6% of the participants. For exercise duration, 39.6% of the surveyed students spent 61 to 90 min per session, with only 5.0% exercising for more than 120 min. Finally, regarding exercise type, 61.3% of the students chose open-skill sports, while 38.7% participated in closed-skill sports.

**Table 5:** The current situation of physical exercise among primary school students.

Exercise frequency			Exercise intensity			Exercise time			Exercise type		
Category	*N*	Percentage (%)	Category	*N*	Percentage (%)	Category	*N*	Percentage (%)	Category	*N*	Percentage (%)
Almost Never	114	6.9	Very Low Intensity	363	22.0	0–30 min	113	6.9	Open Skill Sports	1,011	61.3
1–2 times/week	721	43.7	Low Intensity	362	22.0	31–60 min	512	31.0			
3–4 times/week	587	35.6	Moderate Intensity	461	28.0	61–90 min	653	39.6			
5–6 times/week	159	9.6	High Intensity	437	26.5	91–120 min	288	17.5	Closed Skill Sports	638	38.7
≥6 times/week	68	4.1	Very High Intensity	26	1.6	≥120 min	83	5.0			

Due to the limitations of OLS regression, which is affected by endogeneity and the inherent constraints of the statistical method, it is not able to accurately estimate the robust associations of different exercise frequencies, intensities, times, and volumes on UDVA and KVA. Therefore, this study employs the GPSM method to address potential confounding bias and estimate the dose–response relationships of different exercise frequencies, intensities, times, and volumes on UDVA and KVA. The estimation process was completed in three stages.

First, based on the study’s covariates X , the conditional probability density of the treatment variables—exercise frequency, intensity, time, and volume—is estimated. To satisfy the normality assumption, this study adopts the fractional logit model proposed by Barbara and Marco (2014) to correct the density function. Using the estimation results, the generalized propensity score variable is computed (see Equation 1):


$$\begin{array}{l} E(T_{i}|X_{i})=F(X_{i}\beta )\equiv \frac{\exp(X_{i}\beta )}{1+\exp(X_{i}\beta )},\\ {\hat{R}}_{i}={\left[F(X_{i}\beta )\right]}^{T_{i}}\cdot {\left[1-F(X_{i}\beta )\right]}^{1-T_{i}} \end{array}$$ (1)

Where $T_{i}$ represents the treatment intensity of individual i , $X_{i}$ denotes the covariates of individual i , and F(·) is typically a logit or probit function. $\hat{R}$ is the propensity score for individual i .

Next, a conditional expectation model is established to relate the outcome variable, treatment variable, and generalized propensity score (see Equation 2):


$$\begin{array}{l} E(Y_{i}|T_{i},{\hat{R}}_{i})=\alpha_{0}+\alpha_{1}T_{i}+\alpha_{2}T_{i}^{2}+\alpha_{3}T_{i}^{3}\\ +\alpha_{4}{\hat{R}}_{i}+\alpha_{5}{\hat{R}}_{i}^{2}+\alpha_{6}{\hat{R}}_{i}^{3}+\alpha_{7}T_{i}{\hat{R}}_{i} \end{array}$$ (2)

Finally, based on the estimated function, coefficients are obtained to derive the Average Dose–Response Function (ADRF) for varying levels of exercise frequency, time, intensity, and volume. This allows estimation of the mean values of KVA and UDVA under different exercise conditions, thereby identifying the net causal effect. The estimated function and treatment effect (TE) are as follows (see Equation 3):


$$\begin{array}{c} \begin{array}{l} \mu (t)=\frac{1}{N}\sum_{i=1}^{N}\{{\hat{\alpha }}_{0}+{\hat{\alpha }}_{1}t+{\hat{\alpha }}_{2}t^{2}+{\hat{\alpha }}_{3}t^{3}+{\hat{\alpha }}_{4}\hat{r}(t,X_{i})\\ +{\hat{\alpha }}_{5}\hat{r}{\left(t,X_{i}\right)}^{2}+{\hat{\alpha }}_{6}\hat{r}{\left(t,X_{i}\right)}^{3}+{\hat{\alpha }}_{7}t\cdot \hat{r}(t,X_{i})\} \end{array} \end{array}$$ (3)

Since this study categorizes exercise types into open-skill sports and closed-skill sports, the previously employed GPSM method is not applicable in this case. Instead, this section adopts the 1:1 nearest-neighbor PSM method to estimate the results, ensuring that only data within the common support region of the propensity scores are matched. The treatment variable, exercise type, is defined as a binary variable: samples engaging in open-skill sports are assigned a value of 1, while those not participating in open-skill sports are assigned a value of 0 (0 = closed-skill sports; 1 = open-skill sports). A logistic regression model is used to estimate the probability of each sample receiving the treatment, yielding the propensity score. Subsequently, the nearest-neighbor matching algorithm is employed to select the n most similar untreated samples (i.e., those with the closest propensity scores) as matched counterparts for the treated group. Finally, t-tests are conducted to examine whether the mean values of each characteristic between the treated and untreated groups remain significantly different after matching.

#### 3.2.1 Association between exercise frequency and UDVA, KVA in students

Based on the evaluation steps of the GPS model, propensity scores were calculated and matched on the distribution of “exercise frequency.” To ensure that no significant differences exist in the data characteristics between the treated and control groups after matching, a standard two-sided t-test was used to analyze the balance of the matched data. The balance test results indicated that covariates such as gender, age, outdoor activity time, screen time, as well as control variables including exercise intensity, time, and type, passed the balance test, confirming a good matching effect. Next, exercise frequency was used as the explanatory variable, and the calculated propensity score was used as a control variable to estimate the conditional expectation function of UDVA and KVA.

The dose–response function values corresponding to each exercise frequency were connected by curves, illustrating the association between exercise frequency and UDVA and KVA. Additionally, the standard deviation of the dose–response function values was incorporated to display the upper and lower confidence intervals.

**Figure 2:**
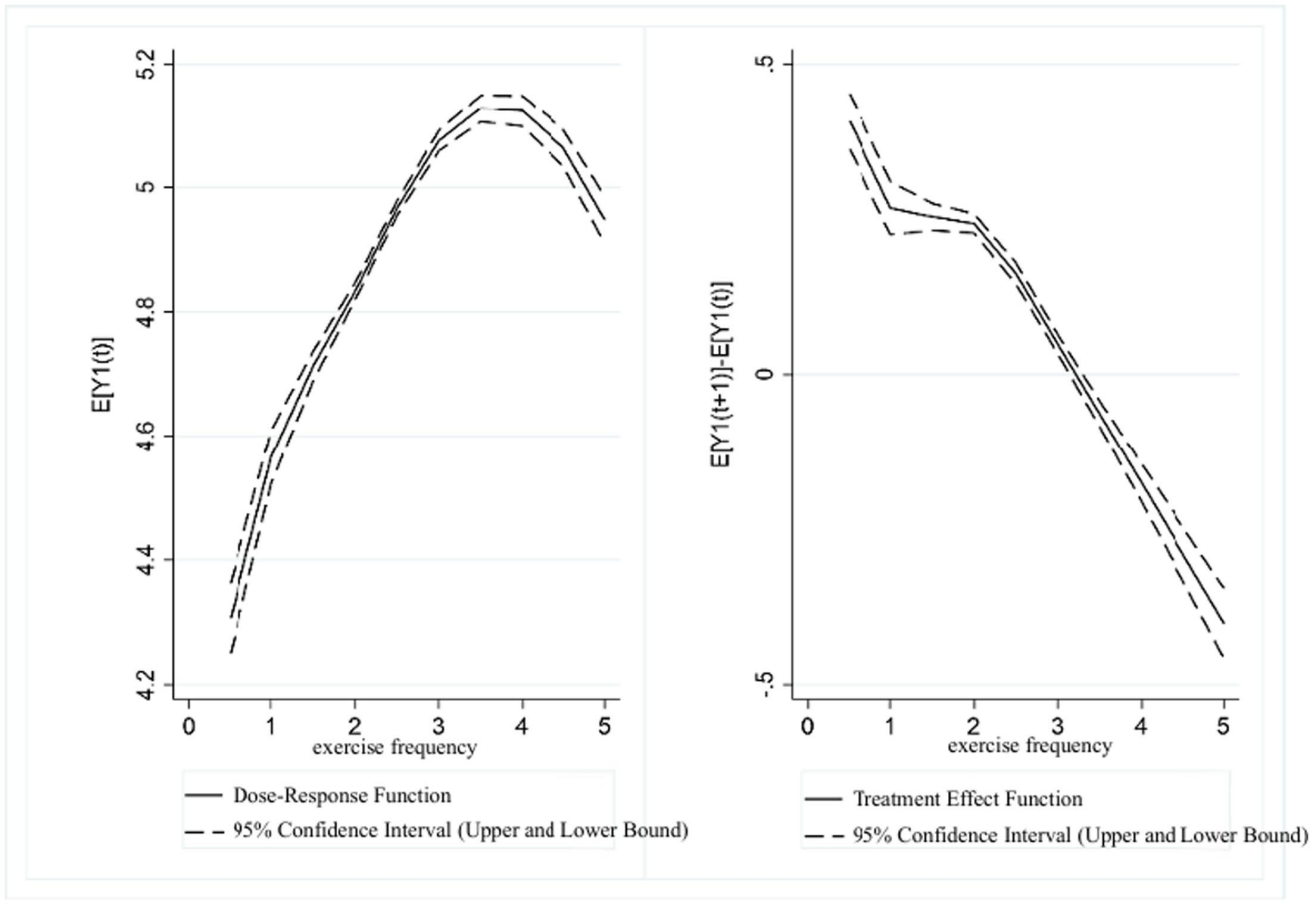
Estimated association between exercise frequency and UDVA in students.

Figure 2 presents the dose–response function and treatment effect function between exercise frequency and UDVA in students. The results showed a “inverted U-shaped” trend between the two variables. In the range of (0, 3.5], higher exercise frequency scores were associated with higher UDVA in students. However, in the range of (3.5, 5], UDVA scores showed a downward correlation with increasing exercise frequency. The treatment effect function of exercise frequency and UDVA indicated that a significant positive association between exercise frequency and UDVA exists in the (0, 3.5] range. In contrast, in the range of (3.5, 5], as the exercise frequency score increased, the positive correlation between physical exercise and UDVA levels gradually diminished.

**Figure 3:**
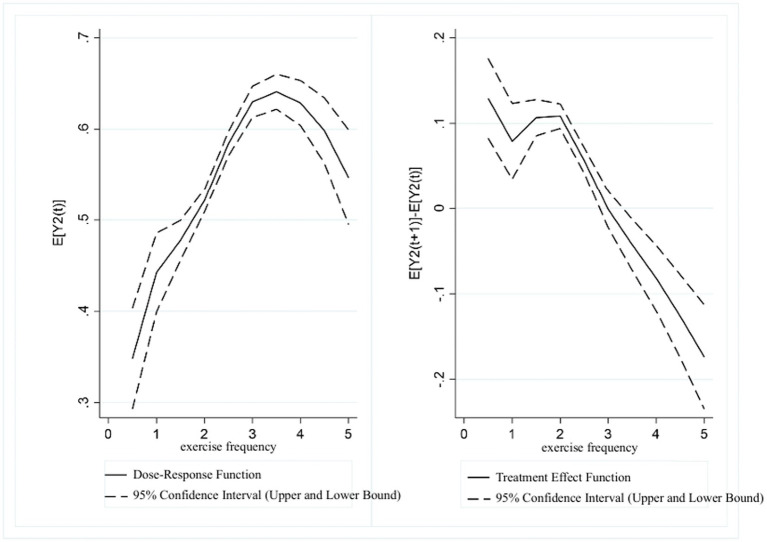
Estimated association between exercise frequency and KVA in students.

Figure 3 presents the dose–response function and treatment effect function between exercise frequency and KVA in students. The results also revealed a “inverted U-shaped” trend. In the range of (0, 3.5], KVA scores were positively correlated with exercise frequency scores. In the range of (3.5, 5], KVA showed a decreasing trend as the exercise frequency score increased. The treatment effect function showed that in the range of (0, 3.5], exercise frequency was significantly associated with higher KVA levels. However, in the range of (3.5, 5], as the exercise frequency score increased, the positive relationship between physical exercise and KVA development gradually declined.

#### 3.2.2 Association between exercise intensity and UDVA, KVA in students

Based on the estimated distribution of “exercise intensity,” propensity scores were calculated and matched. To ensure the balance of data characteristics between the treatment and control groups, a standard two-sided t-test was conducted. The balance test results indicated that covariates such as gender, age, outdoor activity time, screen time, and the variables that needed to be controlled, including exercise frequency, time, and type, passed the balance test, suggesting that the matching process was effective. Exercise intensity was used as the explanatory variable, and the previously calculated propensity scores served as control variables to estimate the conditional expectation functions for UDVA and KVA.

**Figure 4:**
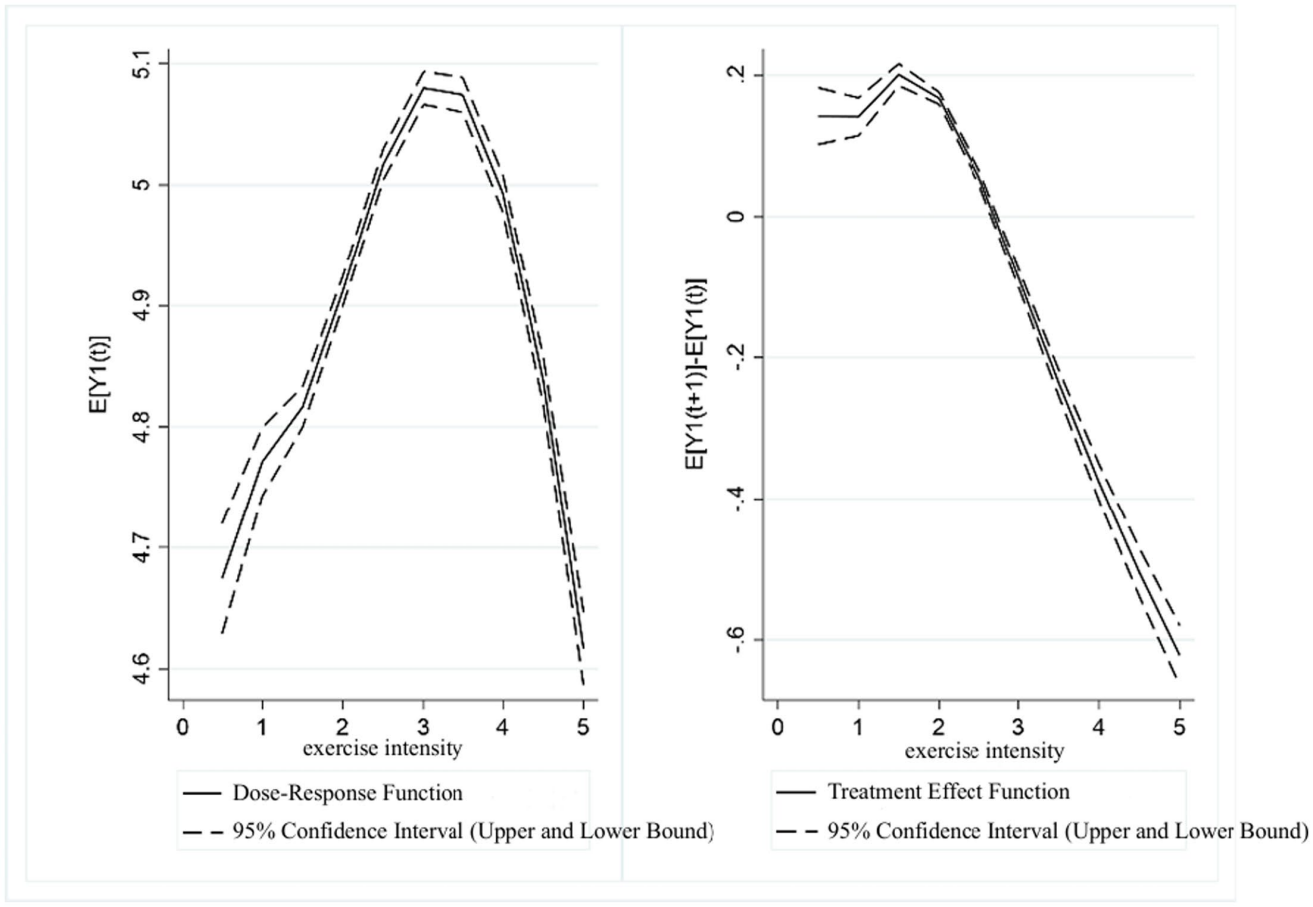
Estimated association between exercise intensity and UDVA in students.

Figure 4 presents the dose–response function and treatment effect function between exercise intensity and UDVA in students. The results revealed a trend of “initial increase, followed by a gradual decrease, and a subsequent significant decrease” in UDVA as exercise intensity increased. In the range of (0, 3], higher exercise intensity scores were associated with a continuous increase in UDVA. However, in the (3, 3.5] range, UDVA scores showed a gradual downward correlation with increasing exercise intensity, which became more pronounced in the (3.5, 5] range. The treatment effect analysis indicated that a significant positive association between exercise intensity and UDVA existed in the (0, 3] range, whereas in the range of (3, 5], the positive correlation between exercise intensity and UDVA levels gradually diminished.

**Figure 5:**
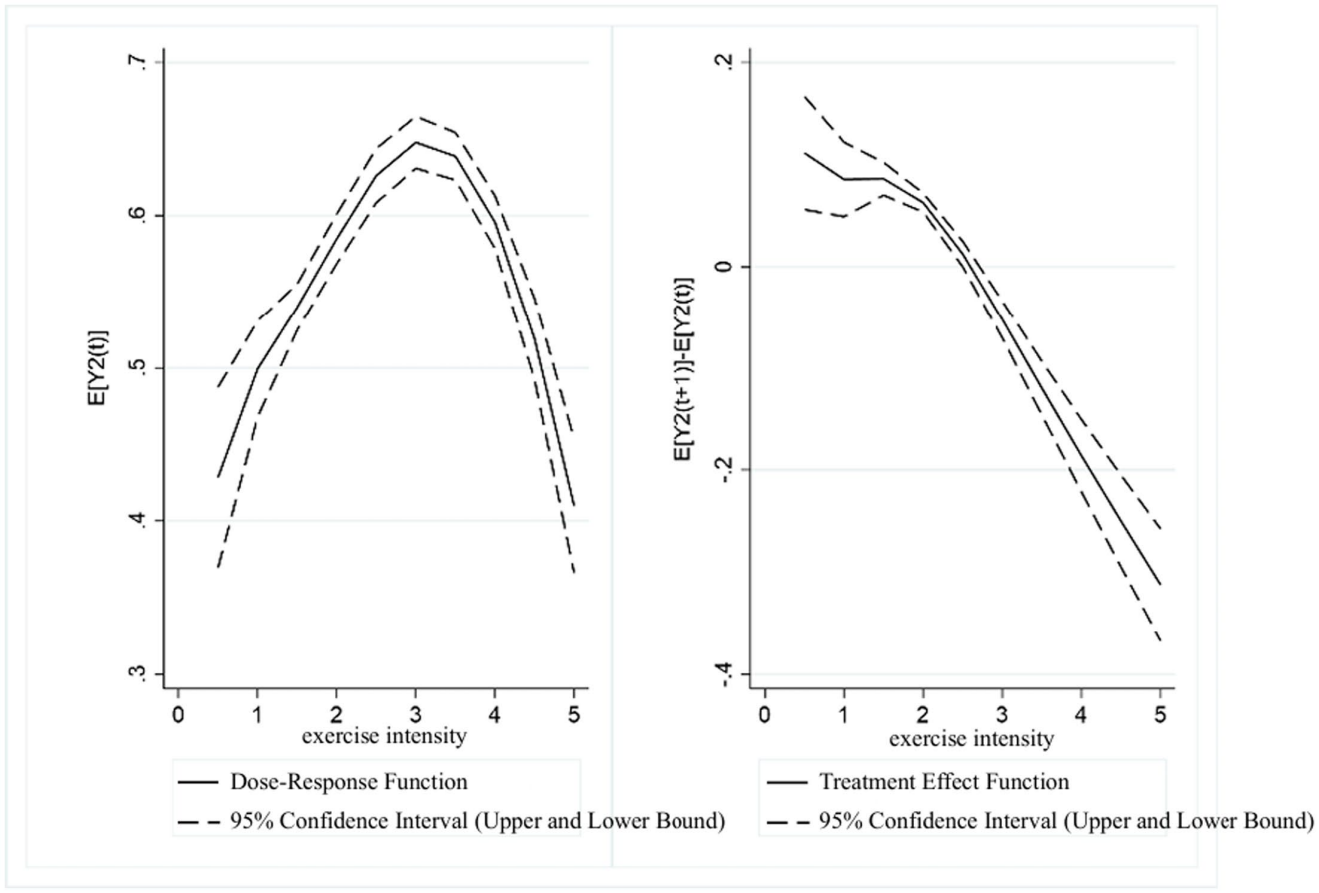
Estimated association between exercise intensity and KVA in students.

Figure 5 presents the dose–response function and treatment effect function between exercise intensity and KVA in students. The results showed an “inverted U-shaped” trend between exercise intensity and KVA. In the range of (0, 3], KVA continuously increased with the rise in exercise intensity. In the (3, 5] range, KVA showed a decreasing trend as the exercise intensity score increased. The treatment effect analysis similarly revealed that in the range of (0, 3], exercise intensity was significantly associated with higher KVA levels. However, in the range of (3, 5], the positive relationship between exercise intensity and KVA development gradually declined as the exercise intensity score increased.

#### 3.2.3 Association between exercise time and UDVA, KVA in students

Based on the estimated distribution of “exercise time,” propensity scores were calculated and matching was performed. To ensure that there were no significant differences in data characteristics between the treatment and control groups after matching, a standard two-sided t-test was used to analyze the balance of the matched groups. The balance test results indicated that covariates such as gender, age, outdoor activity time, screen time, and control variables such as exercise frequency, intensity, and type, were well balanced, suggesting a successful matching process. Exercise time was used as the explanatory variable, and the previously calculated propensity scores were included as control variables to estimate the conditional expectation function for UDVA and KVA.

**Figure 6:**
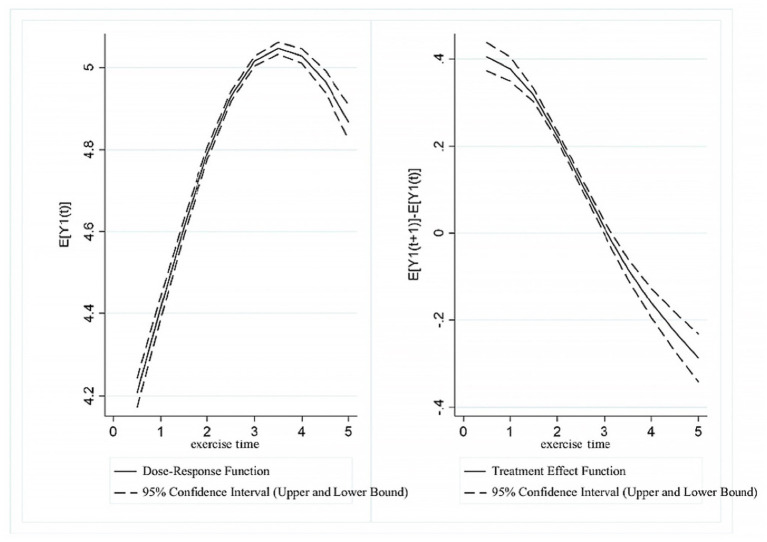
Estimated association between exercise time and UDVA in students.

Figure 6 presents the dose–response function and treatment effect function for UDVA in students with respect to exercise time. From the dose–response function graph, it is evident that there was a significant “inverted U-shaped” relationship between exercise time and UDVA, with the inflection point occurring around a value of approximately 3.5. Specifically, in the range of (0, 3.5], higher exercise time scores were associated with higher UDVA in students. However, in the range of (3.5, 5], UDVA scores showed a downward correlation with increasing exercise time. The treatment effect function indicated that a significant positive association between exercise time and UDVA existed in the (0, 3] range. In contrast, within the (3, 5] range, as the exercise time score increased, the positive correlation between exercise time and UDVA levels gradually diminished.

**Figure 7:**
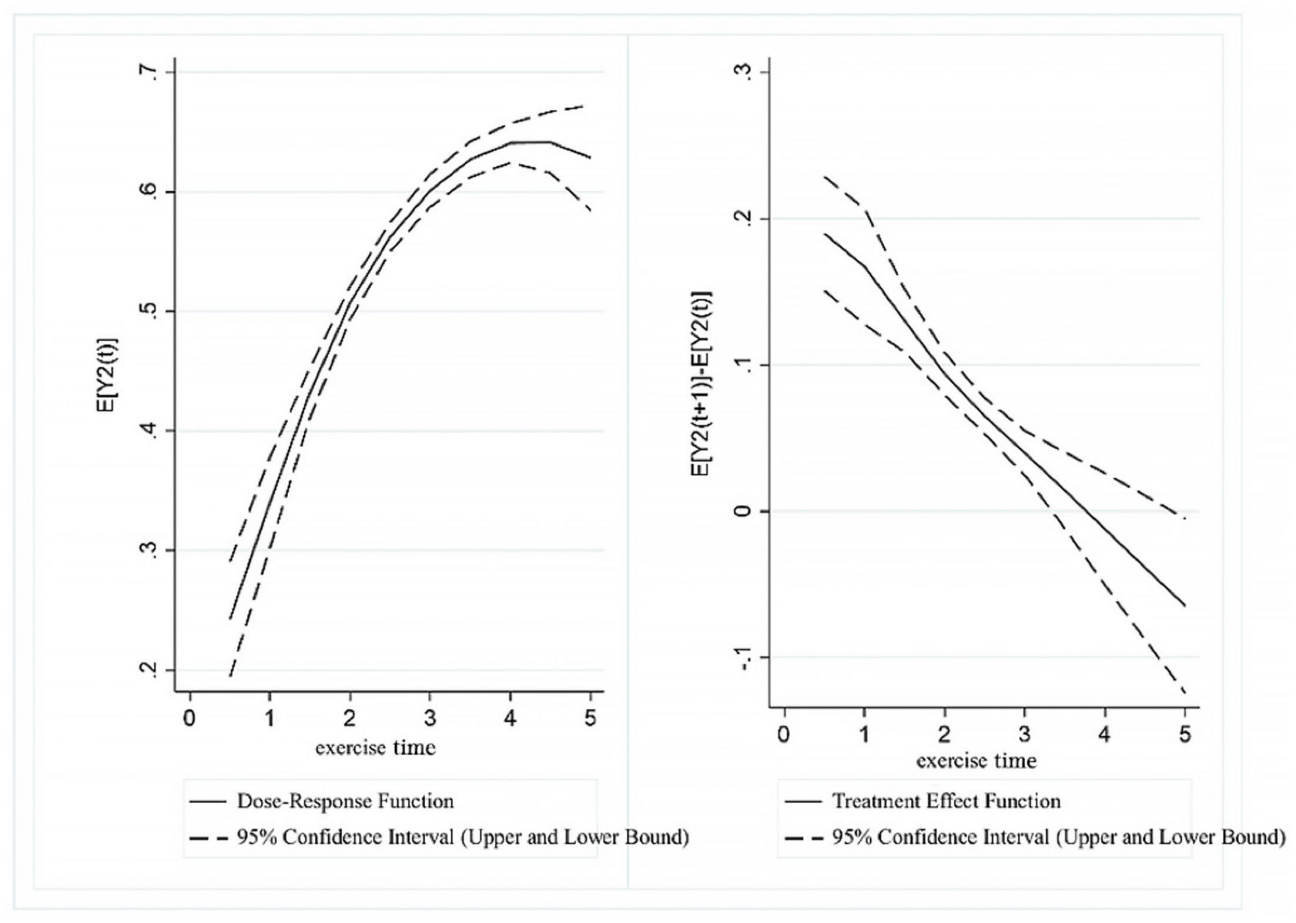
Estimated association between exercise time and KVA in students.

Figure 7 presents the dose–response function and treatment effect function for KVA in students with respect to exercise time. From the dose–response function graph, it is apparent that when exercise time was in the range of (0, 4], KVA scores were positively correlated with exercise time scores. In the (4, 5] range, however, KVA showed a decreasing trend as the exercise time score increased. The treatment effect function for exercise time and KVA similarly exhibited a rising and then falling trend. Specifically, in the range of (0, 4], exercise time was significantly associated with higher KVA levels. However, in the (4, 5] range, the positive relationship between exercise time and KVA development gradually declined as the exercise time score increased.

#### 3.2.4 Association between exercise volume and UDVA, KVA in students

Based on the estimated distribution of “exercise volume,” propensity scores were calculated and matched. To ensure that there were no significant differences in data characteristics between the treatment and control groups after matching, a standard two-tailed t-test was used to analyze the balance of the matched groups. The balance test results showed that covariates such as gender, age, outdoor activity time, and screen time, passed the balance test, indicating that the matching process was effective. Exercise volume was used as the explanatory variable, with the previously calculated propensity score as a control variable, to estimate the conditional expectation functions for UDVA and KVA.

**Figure 8:**
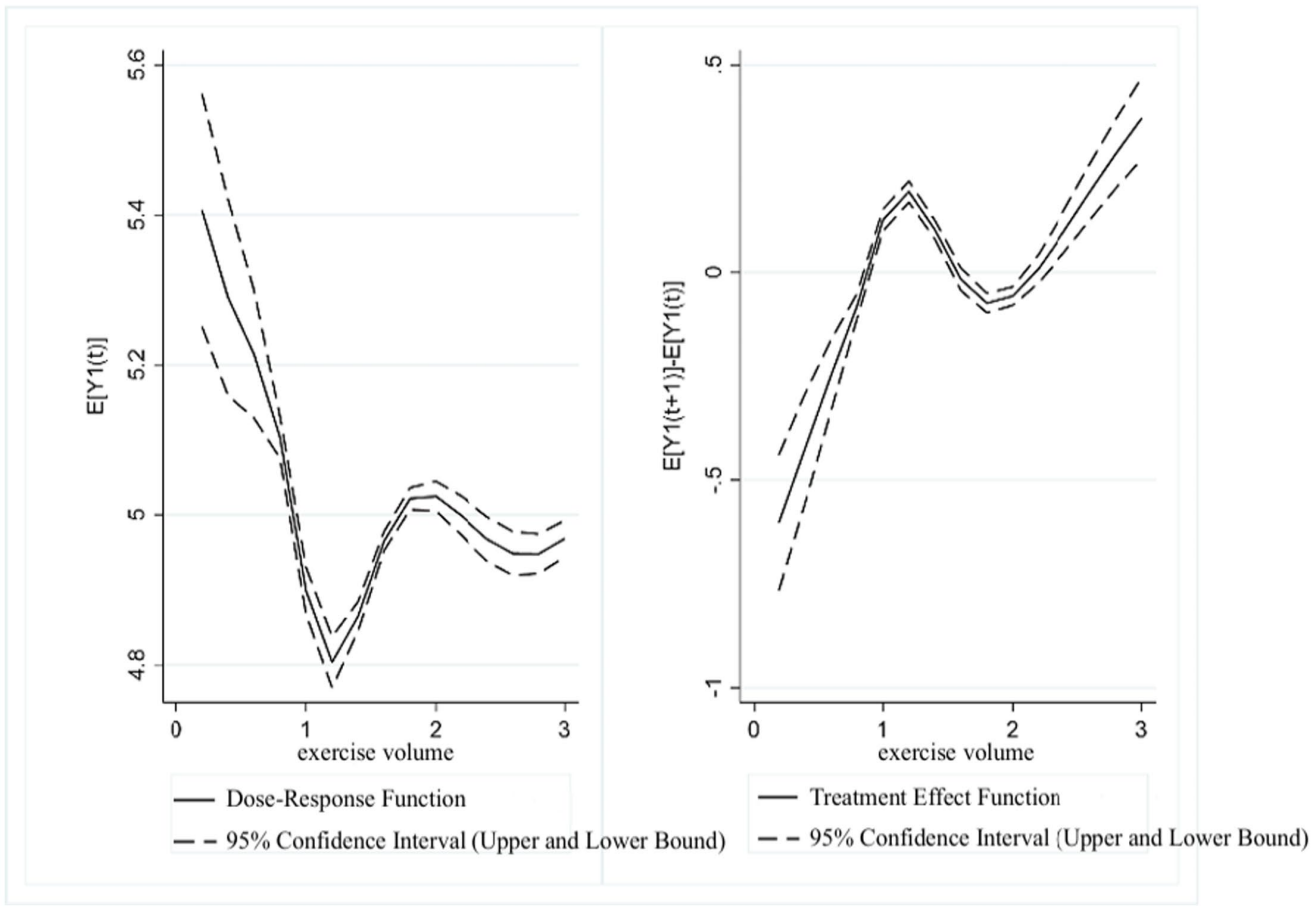
Estimated association between exercise volume and UDVA in students.

Figure 8 presents the dose–response function and treatment effect function of exercise volume and UDVA in students. The results showed that in the range of (1.2, 2], higher exercise volume scores were associated with a continuous improvement in students’ UDVA. However, in the range of (2, 3], as exercise volume continued to increase, UDVA scores showed a gradual downward correlation. The treatment effect function revealed that a significant positive association between exercise volume and UDVA existed within the (0, 1.5] range. In the (1.5, 2] range, this positive correlation gradually weakened, while in the (2, 3] range, a positive relationship between exercise volume and UDVA was again observed.

**Figure 9:**
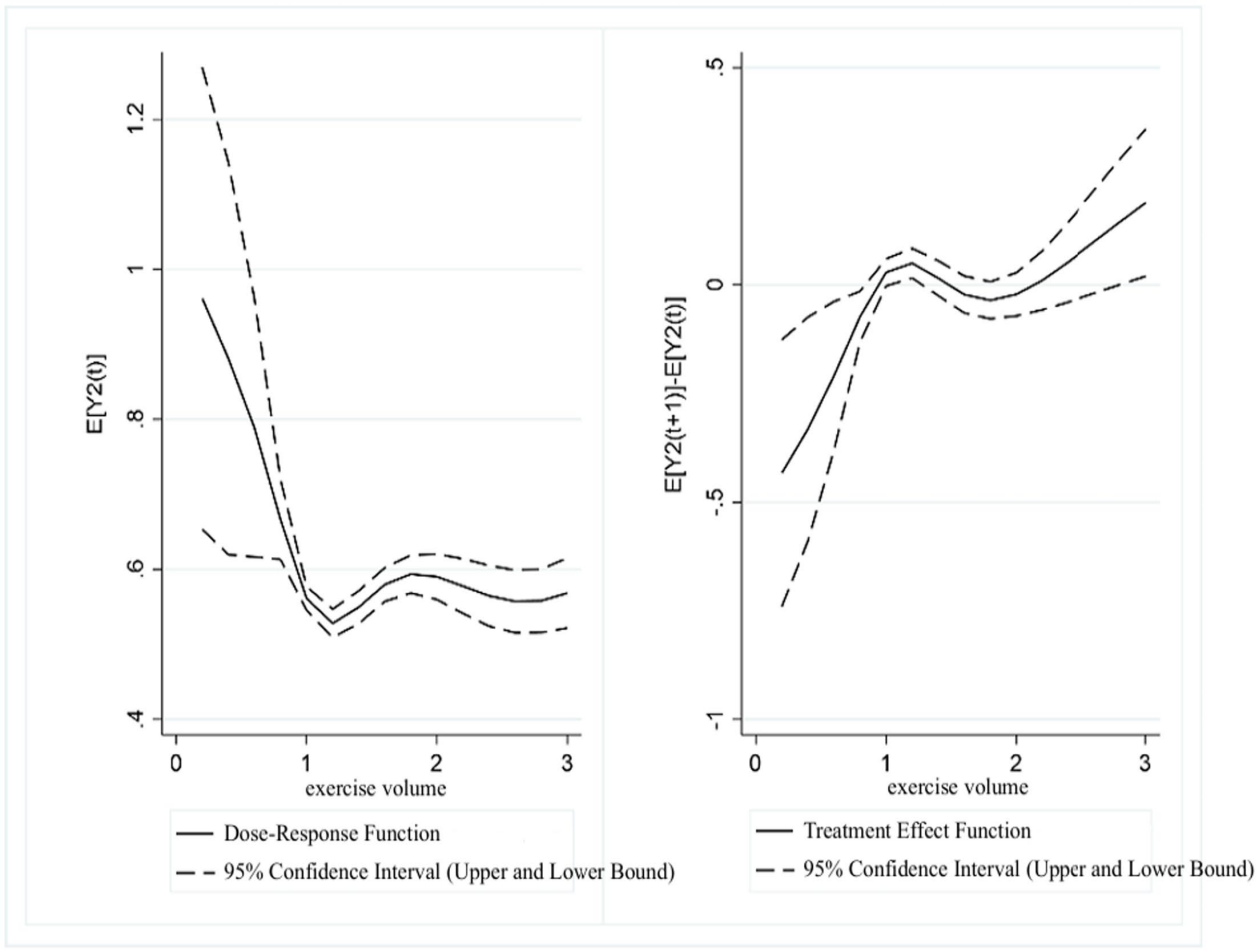
Estimated association between exercise volume and KVA in students.

Figure 9 presents the dose–response function and treatment effect function of exercise volume and KVA in students. The results showed that in the range of (1.2, 1.8], KVA scores were positively correlated with exercise volume scores. However, in the (1.8, 3] range, KVA shows a decreasing trend as the exercise volume score increased. The treatment effect function analysis indicated that in the range of (0, 1.5], exercise volume was significantly associated with higher KVA levels. In the (1.5, 2] range, this positive association gradually diminished, and in the (2, 3] range, the results again indicated a positive relationship between exercise volume and KVA development.

#### 3.2.5 Association between exercise type and UDVA, KVA in students

The balance check for propensity score matching (Table 6) showed that after matching, the pseudo-R^2^, chi-square, mean bias, and B-values all decreased substantially, with the standardized bias for all variables being less than 10%, indicating that the differences between the treatment and control groups were reduced, and the sample matching balance was satisfactory.

**Table 6:** Results of matching balance assumption test.

Matching method	Matching stage	Pseudo-*R*^2^	Chi-square	Mean bias (%)	*B*-value (%)	*R*-value
Nearest Neighbor Matching (1:1)	Pre-matching	0.072	127.64	15.7	64.1*	0.70
	Post-matching	0.007	14.31	2.9	8.5	1.04

B > 25% or 0.5 ≤ *R* ≤ 2 is marked with *.

As shown in Table 7, after propensity score matching, the ATT effect value for UDVA was statistically significant (*p* < 0.01), indicating a significant difference between open-skill and closed-skill sports in their association with UDVA among primary school students. The ATT effect value was 0.0374, suggesting that participation in open-skill sports was linked to a more positive level of UDVA. In contrast, the ATT effect value for KVA was not statistically significant (*p* > 0.05), implying that there was no significant difference between open-skill and closed-skill sports in their relationship with KVA.

**Table 7:** Matched results for uncorrected distance visual acuity and kinetic visual acuity.

Variable	Indicator	Treatment variable	Control variables	Coefficient	Standard error	*T*-value
UDVA	Pre-matching	4.9641	4.8387	0.1254	0.0115	10.90
	ATT	4.9620	4.9246	0.0374	0.0191	1.96**
	ATU	4.8612	4.9244	0.0857		
	ATE			0.0557		
KVA	Pre-matching	0.5754	0.5442	0.0312	0.0116	2.69
	ATT	0.5716	0.5930	−0.0215	0.0184	−1.17
	ATU	0.5518	0.5558	0.0040		
	ATE			−0.0118		

*p < 0.05, **p < 0.01, ***p < 0.001.

## 4 Discussion

The results of this study indicated that while gender does not significantly influence UDVA levels, boys performed significantly better than girls in KVA (*p* < 0.05). This suggested that while static visual acuity was similar across genders in this population, dynamic visual functions may be more sensitive to gender-related biological or behavioral variations. With the continuous advancement of digital technology and the widespread use of electronic devices such as computers, televisions, smartphones, and tablets in households, the time spent on near-distance visual tasks by students has generally increased. This has led to a gradual narrowing of the gap in near-distance eye usage between genders, which in turn reduces the disparity in visual acuity levels between boys and girls (19). In addition, significant differences in UDVA and KVA were observed across different grade levels. Regarding UDVA, a clear trend of gradual decline was found across the surveyed grade levels. The mean UDVA of second-grade students was higher than that of third-grade students, which was in turn higher than that of fourth-grade students, and finally, the fifth-grade students had the lowest average UDVA. This trend aligns with the current national prevalence of UDVA decline among primary school students and is consistent with the findings of Zhao et al. (20). Regarding KVA, fourth-grade students exhibited significantly better performance than students in other grades. This finding corroborates related research. Yang et al. conducted a comparative analysis of the visual acuity status of primary school students in China and Japan and found that KVA in Chinese children develops slowly before the age of 8–9, peaks around the age of 9, and then starts to decline after that (21).

The results of this study revealed that the association between exercise frequency and both UDVA and KVA was not a simple linear correlation. Instead, a “inverted U-shaped” relationship was observed. As the frequency of physical exercise increased, the positive association between exercise on UDVA and KVA levels gradually strengthened. This trend continued until the frequency reached 3 to 4 times per week, at which point students’ UDVA and KVA were linked to their optimal levels. This finding is consistent with previous research. Most scholars believe that engaging in physical exercise at least three times a week can effectively improve children’s visual health (22, 23). Children and adolescents who participate in physical exercise regularly tend to maintain better visual acuity levels. Furthermore, exercise has also been shown to exert a certain degree of improvement in individuals who are already myopic (24). This is primarily because physical exercise helps to relax the ciliary muscle. Additionally, exercise promotes emmetropization by enhancing choroidal blood circulation (25). During adolescence, the eyeball is still in the process of development. However, with the widespread use of electronic devices and increasing academic pressure, prolonged near work has become common. This sustained near work forces both the ciliary muscle and extraocular muscles to remain in a highly tense state, eventually leading to ciliary muscle spasm and, subsequently, myopia. Regular participation in physical exercise can effectively alleviate this condition. By promoting relaxation of the eye muscles, exercise contributes to the improvement of visual acuity in children and adolescents.

Regarding exercise intensity, the results of this study indicated that moderate intensity exercise showed the strongest positive association with students’ vision. Wang also reported a significant association between the duration of moderate-intensity physical activity and the risk of poor vision. Engaging in moderate-intensity physical activity was shown to effectively protect students’ vision (26). This protective effect is attributed to the improvement in ocular muscle function and blood circulation during exercise. After exercise, intraocular pressure decreases, while choroidal blood flow velocity increases, ensuring adequate blood supply to the retina. Higher levels of retinal blood supply have been found to promote the development of ocular nerves and muscles in children (27). Wang et al. investigated the effects of aerobic exercise at different intensities on intraocular pressure in female university students with moderate to high myopia. Their findings showed that moderate-intensity aerobic exercise led to more pronounced reductions in intraocular pressure among students with emmetropia and moderate myopia (28). In contrast, excessive exercise intensity can induce negative changes in neutrophil and NK cell function, as well as in salivary immunoglobulin A (IgA) and certain types of inflammatory macrophages, ultimately impairing immune function (29). Compromised immune function increases the risk of various ocular complications, ranging from mild symptoms to vision-threatening conditions (30). Therefore, engaging in moderate-intensity physical exercise to maintain appropriate intraocular pressure is crucial for protecting vision.

Regarding exercise time, the results of this study revealed a positive association between the length of exercise and both UDVA and KVA in students. When the exercise time reached 61 to 90 min, the positive association with students’ UDVA was most pronounced. In contrast, the most favorable KVA levels were observed when students exercised for 91 to 120 min. Song et al. conducted an intervention with a 90-min exercise session and found that students in the experimental group showed improved visual acuity (31). Similarly, Gong et al. reported that children and adolescents who engaged in at least 60 min of physical activity per day demonstrated better visual health than their peers with lower levels of physical activity (32). Existing research also suggests that, in addition to regular physical activity, increasing outdoor physical activity by 3 h per week or 6 h per week can further enhance visual acuity in primary school students (33, 34). Among these, an additional 6 h of outdoor activity per week was shown to be particularly effective. This improvement can be attributed to the exposure of the eyes to natural outdoor light, which stimulates the production and release of dopamine in the retina. This process triggers downstream signaling pathways and enhances scleral fiber strength (35), thereby slowing axial elongation. This finding is consistent with the results reported by Li et al., who conducted a one-year outdoor exercise intervention and monitored both myopia progression and axial length changes among primary school students. Their study demonstrated that increasing outdoor activity time effectively controlled axial elongation and the progression of myopia, playing a key role in preventing and slowing down the onset and development of myopia in primary school students (36). While we did not measure axial length, our findings regarding the positive correlation between exercise time and both UDVA and KVA provide functional evidence that aligns with these structural observations.

Regarding exercise volume, the results of this study indicated that a moderate amount of exercise showed a stronger positive association with both UDVA and KVA of students. This finding aligns with previous research. Yao et al. demonstrated in their experiment that moderate exercise volume exerted positive and beneficial effects on both UDVA and KVA (37). Insufficient physical activity has been identified as a key factor contributing to the decline in physical fitness and the deterioration of visual health among primary school students. Ma found that indicators such as endurance, speed, strength, and vital capacity among primary and secondary school students exhibited a declining trend year by year, while the prevalence of obesity and poor vision continued to rise. This study highlighted insufficient physical activity as one of the main reasons for the declining physical fitness of Chinese students (38). With the accelerating pace of modern life and increasing academic competition, primary school students face multiple challenges, including insufficient sleep, excessive near work, and a lack of interest and awareness regarding physical exercise. These factors exacerbate both physical and psychological burdens, indirectly contributing to the decline in overall physical fitness. Physical activity helps alleviate eye fatigue by promoting metabolism and enhancing ocular blood circulation. It also strengthens the contraction intensity and regulatory capacity of both the intraocular ciliary muscles and the extraocular convergence muscles, enabling better coordination between accommodation and ocular alignment (22). During physical activity, the visual tasks that require alternately tracking near and distant objects can effectively activate the contraction and relaxation of the ciliary muscles. This process helps relieve muscle spasms, enhances kinetic visual acuity, improves uncorrected distance visual acuity, and ultimately contributes to better overall vision (10).

This study also found that open-skill sports showed a more pronounced positive association with UDVA in students compared to closed-skill sports, whereas no significant divergence was observed between the two types of sports in terms of their relationship with KVA. Yin et al. suggested that both open-skill and closed-skill sports involve visual tracking of operational targets during movement, with alternating shifts between near and distant vision providing effective training for the ciliary muscles (16). For instance, in ball sports regardless of whether the ball is large or small the ball serves as a target for visual tracking. Similarly, in activities such as Tai Chi or aerobics, movements like tracking hand movements with the eyes also facilitate alternating adjustments of the ciliary muscles, contributing to the development of visual function. Jin et al. designed physical activities incorporating both open-skill and closed-skill movement tasks aimed at enhancing KVA and conducted an experimental intervention. The results indicated that both types of activities improved UDVA and KVA in students with myopia, and these effects were not influenced by gender (17). However, open-skill sports generally require more frequent visual adjustments and target tracking, resulting in higher levels of visual interaction. This intensive visual engagement plays a crucial role in promoting ciliary muscle health, thereby exerting a stronger positive effect on UDVA. In contrast, closed-skill sports involve relatively simpler and more repetitive movements, with fewer shifts between near and distant vision, leading to less ciliary muscle activation and flexibility adjustment, thus limiting their impact on UDVA. This is consistent with findings from previous cross-sectional studies, which revealed that athletes participating in ball sports exhibited superior functional vestibulo-ocular reflex and proprioception compared to those participating in combat sports, indicating that engagement in ball sports may be more beneficial for enhancing athletes’ visual and sensory system functions (18).

It should be noted that the outcome variables in this study, UDVA and KVA, primarily reflect functional visual performance rather than the refractive status or axial length of the eye. While our findings demonstrated a positive association between physical exercise and visual acuity, these results should be interpreted as improvements in visual function. Future research incorporating refractive error measurements is needed to further clarify the direct implications for myopia incidence and progression.

## 5 Conclusion

In this cross-sectional observation among students in Grades 2 to 5 in Suzhou, UDVA exhibited a gradual decline with increasing grade levels, whereas KVA peaked in Grade 4 and subsequently declined. Engaging in physical exercise 3 to 4 times per week at moderate intensity and volume corresponded to the relatively highest observed values for both UDVA and KVA in students. The highest statistical associations for UDVA were linked to an exercise time of 61 to 90 min per session, while 91 to 120 min per session was associated with the highest KVA scores. Additionally, open-skill sports showed a more pronounced positive relationship with the healthy levels of UDVA in students.

## 6 Shortcomings and prospects

This study has several notable strengths, including a relatively large sample size and the concurrent evaluation of both UDVA and KVA. By employing the FITT framework to categorize physical activity and utilizing advanced statistical methods like GPSM and PSM to address confounding, we were able to approximate robust dose–response patterns. However, several limitations should be acknowledged: (1) Due to constraints in human and material resources, the survey was conducted only in selected primary schools in Suzhou, and regional differences in students’ physical activity patterns and other vision-related factors may exist, it may limit the external validity of the findings. Future research should expand the geographical coverage and increase the sample size, and implement a multi-center design with a more diverse sampling frame to capture these cross-regional variations. In addition, a formal *a priori* power analysis (e.g., using G*Power) was not conducted, which may limit the statistical rigor of the study. (2) Regarding the empirical strategy, the treatment variables (exercise frequency, intensity, etc.) were treated as continuous in the GPSM analysis, although they are inherently ordinal. While this allowed for the estimation of a continuous dose–response function, it may affect the precision of the statistical inference. Furthermore, since the sample was drawn from multiple schools and classes, potential clustering effects (intra-group correlations) may exist. Future studies could employ more advanced statistical techniques, such as multi-level modeling or cluster-robust standard errors, to further refine the precision of the results. (3) In terms of vision health indicators, this study assessed only UDVA and KVA, which reflect functional visual performance rather than the refractive status itself. Therefore, the findings should not be directly extended to myopia incidence or progression. Future studies should incorporate a broader range of indicators, such as refractive error (diopters) and axial length, to provide a more comprehensive understanding of visual health. Finally, future research could employ longitudinal or experimental designs to better clarify the causal relationship between physical activity and vision. (4) Furthermore, although GPSM was employed to mitigate the influence of confounding variables, it is important to acknowledge that the identification of true causal effects relies on several strong statistical assumptions—including no unmeasured confounding, correct model specification, and sufficient overlap. As these assumptions cannot be fully verified in a cross-sectional observational study, the results of this study should be interpreted as estimated dose–response associations rather than definitive causal relationships.

## Data Availability

The raw data supporting the conclusions of this article will be made available by the authors, without undue reservation.
